# The child everyone has inside: anthropology and the labor theory of value

**DOI:** 10.1007/s10624-022-09649-6

**Published:** 2022-02-05

**Authors:** Luis F. Angosto-Ferrández

**Affiliations:** Department of Anthropology, University of Sydney, Sydney, NSW Australia

**Keywords:** Anthropology, Labor theory, Value, Marx, Engels, Essential work, COVID-19

## Abstract

This paper revisits the debate on the relevance of the labor theory of value for the anthropological task. It argues that the labor theory of value can creatively inform and reformulate in critical ways a variety of social issues addressed through anthropological lenses. The argument is sustained by two main exercises: first, a critical overview of the foundations of the labor theory of value outlines the reasons why it opened new grounds for anthropological and, more generally, for social-scientific enquiries. Second, a discussion of the key points of friction between scholarship that attempts to develop an “anthropological” theory of value as an end in itself and anthropological scholarship that resorts to the (labor) theory of value to critically inform research.

Every now and then, a theoretical abstraction hits us with the strength of a boxing champion. This is one of those times. We are all experiencing the multilayered effects of the COVID pandemic in partly dissimilar forms, but in parallel we all realize that these diverse experiences are linked by a common denominator: the “work” that keeps our societies going. This abstraction is in everyone’s minds these days. Indeed, it seems that this pandemic has brought out the “child” we all have inside.

I am of course referring to the “child” that featured in a well-known communication between Marx and his friend Kugelmann (Marx 2000 [1868]: 563). This was a child who, unlike selectively forgetful adults, understood all too well the premise that “a nation which ceased to work [even if it were for a short period of time] would perish” (ibid.). For Marx, such recognition of the fundamental process that sustains (and makes) social life was shrouded in theoretical implications, to the extent that he presented that premise as the grounding of “natural laws” (ibid.).

The laws to which Marx referred were of course social, not “natural.” His point was that, necessarily, it is in any society’s “nature” to establish (historically specific) forms of distributing the “total labor” required to meet social needs. He was rather scornful about critics of his conceptualization of value who disregarded that apparently simple premise in their analyses.

Incidentally, what we are witnessing amidst this pandemic is that, beyond any abstractions, people demonstrate in multiple ways how essential a place those “natural laws” Marx referred to occupy both in their lives and in the ideas they have about those lives. Politicians and epidemiologists, workers and capitalists, women and men, racialized and non-racialized people, elders and youngsters, migrants and “expats,” the unemployed and the retired, the believer and the faithless, the underemployed and the idle rich—you name them, beyond any binaries. Everyone is letting their inner child out, as it were: everyone is acting, speaking and gaining insights into their personhood and their positions and roles in society in relation to that “childish” premise.

From the tribunes of expertise or from the standpoints of common sense, most different people appeal to the necessity of balancing the care for life (“public health”) and work (or “the economy”). It is immediately evident that no natural equilibrium exists in that equation: starkly different opinions are passed on the actual, and the ideal, point of balance. Yet nevertheless many people respond to the call, be it in the name of social responsibility, under the coercion imposed by the lack of alternatives, or under a combination of both drives: “work” must go on.

This phenomenon constitutes a collective acknowledgment of “work.” Nonetheless, we find dramatic manifestations of the dissimilar experiences that this abstraction generates among people in different social positions partly conditioned by the (politically sanctioned) norms that regulate and make specific distributions of “total labor” effective in society.

An examination of the category “essential work” is revealing in this respect. This category, so rapidly normalized in public discussion and policy design amidst the pandemic, has in practice encompassed different types of jobs in different societies,^1^ often just involving a change in the denomination of labor that, sensu stricto, was already indispensable for our societies to function before the pandemic (Guerrero et al 2020). That variability in the definition of “essential work” in different societies does not depended as much on the existence of “culturally” diverse priorities in different societies as it has on the political character of the decision-making process behind such categorization. In any society, the social process of categorizing laborers is “power-laden” (Carbonella and Kasmir 2014: 7). In practice, different social actors have unequal capacities to lobby and influence political elites and governments in support of their definitions of priorities, and amidst the current pandemic those capacities proved critical when it came to identify which areas of the economy and what services are “essential”—generally favoring large businesses (Trnka 2020: 11).

These factors are consequential for everybody’s lives, and exploring them a bit further will help us set background to this introduction. In order to do so I will focus on social dynamics that led to and were sparked by the categorization of essential work in Spain, my country of origin and an exemplar case of a society that, as of this writing, remains severely affected by the multilayered effects of the pandemic.

## The laws of “work”: a common process that diversifies

In Spain, “essential workers” include employees of the public healthcare system and seasonal agricultural workers, representing two sectors in which labor has been deemed indispensable for society. The forms and functions of the labor undertaken by these groups of workers are obviously different: one group provides labor in a service of care so people can live as healthily as possible; the other group provides labor to agricultural production so people can eat and live (not necessarily the people in the country where that production takes place, given the focus on exportation of much of that production).

The process that led to the government-sanctioned categorization of such types of labor as essential was markedly different too. In Spain, where social consensus around the principles of the welfare state in relation to healthcare provision remains comparatively strong across classes even in times of “austerity” (i.e., structural adjustments of neoliberal orientation [Powers and Rakopoulus 2019]), public healthcare work was categorized as essential from the outset—somewhat by default. On the contrary, the categorization of agricultural work as “essential” resulted from the mobilization of a variety of political actors and organized business-owner lobbies that demanded specific regulatory conditions. Organizations of landowners and large agricultural producers campaigned strongly as soon as confinements and border restrictions were introduced by the national government in March 2020. They contended that a shortage of labor (largely dependent on temporary foreign workers in their sector) would have devastating effects on the spring/summer harvests. “We need 120,000 people we do not have,” commented a spokesman of the Coordinadora de Organizaciones de Agricultores y Ganaderos (COAG), estimating a 40% shortage in seasonal labor for the sector. The European Commission, a political organ of the European Union (EU) that is notably responsive to the lobbying of organized groups of big business owners and producers in sectors deemed strategic for the regional interest, had already made public recommendations in this regard: agricultural production had to be protected. The Spanish government responded swiftly to these stimuli: on April 7, 2020, a decree was passed with “urgent” regulations for agricultural employment (BOE 2020) that flexibilized conditions of employment, including the possibility that foreign immigrants without working permits could be hired.

Differences in the process that led to their categorization as “essential” workers, as well as the differences in the forms and functions of the labor they undertake, did not preclude that both healthcare and agricultural workers experienced some similar circumstances as laborers who have to keep “work” going. First, due to the specific conditions in which they live and work (or how the conditions of their work shape the way in which they can live), among both sets of workers have been high numbers of infections. Second, because experiences of exploitation and stress have become even more acute for many of them precisely as they have been performing “essential work,” and as a result members of both groups have undertaken protests in demand of better working conditions. And yet again, further examination of these commonalities soon reveals how social difference between these workers is (re)produced through the forms in which the society they work in distributes and hierarchizes their labor. This socially produced difference contributes to explain why the experiences that people have of their common “essential” character in society can be most dissimilar, as has been the public recognition these two groups of workers have received from other members of society.

For example, among the health sector workers, the risks of infection are markedly higher while they are on duty, in the premises where they work; for them, “getting home” can be associated with a significant degree of protection against infection. On the contrary, among the agricultural workers, higher risks came before and after their waged work took place: generally crowded and poorly serviced accommodation and transportation are in the background, making “getting home” an extension of the risk of infection.

In terms of social recognition, workers in the public healthcare system received daily signs of symbolic support in the form of rounds of applause for many weeks, and particularly during the initial period of government-fostered confinement: at 8 pm, hundreds of thousands of people synchronized this show of recognition throughout Spain, coming out onto their balconies and windows, or stepping out at the front of their houses. On the contrary, the recognition of the “essential” agricultural workers was marginal. A few associations and political groups took the opportunity to denounce the precarious situation of these workers, who in addition to low wages endure extremely harsh conditions of work (ranging from the lack of adequate protocols to protect them from heat/cold and exhaustion in the long days of work to unequipped and often unhealthy living quarters). But that supportive activism contrasted with the cases in which those very same agricultural workers, most of them migrants from African, Latin American, and Eastern European countries, received overt hostility from people who scapegoated them as responsible for the spread of COVID-19 infections in the locations where they work. Those accusations revealed the divergent assessment criteria with which some members of society evaluate the performance of different types of “essential workers.”

Evidence of the ways in which the common foundation of “work” is a source of consequential diversification is found in the protests these two groups of workers are undertaking during the pandemic. This provides a crystallized example of how people express politically the way in which they perceive and experience this social diversification in culturally mediated forms.

In October 2020, the National Confederation of Medical Sector Unions (Conferación Estatal de Sindicatos Médicos, CESM) called for monthly national level strikes, starting on the 27th of that month. The last comparable national-level industrial action among doctors took place 25 years ago—an indication of how exceptional the current scenario is in the own view of doctors. Doctors complained about “insufficient financing for the sector” and about the “abuse of casualization of work in the management of personnel,” among other things (Plaza Casares 2020a [my translation]). In the background of these demands is the disinvestment that the public health system experienced in the past decade, severely affected by the austerity policies applied by successive Spanish governments (of neoliberal orientation) as the effects of the so-called Great Financial Crisis started to strike the country (Heras-Mosteiro et al. 2016; Cervero-Liceras et al. 2015).

Among temporary agricultural workers, the heightened experience of exploitation also gave rise to collective protests during these months. Like the doctors, agricultural workers complained about their working conditions in general, and about the lack of adequate protections to undertake “essential work” amidst a spreading virus in particular. But, with far more obstacles impeding their unionization and with far less bargaining power as a result,^2^ their protests were localized rather than coordinated at a national level, and took forms that were different from the protests of the doctors.

In some journalistic headlines those protests were labeled as “rebellions” and “mutinies,” a discursive denomination that projected the distinction that some journalists and part of the citizenry make between racialized foreigners and non-racialized nationals. But, in parallel, another question transpired through that categorization: the terms used in those journalistic reports (rebellion, mutiny) were more evocative of protests among slaves and coerced laborers than of protests among free workers under voluntarily established contractual relations—a key ideological premise of liberal capitalism. The thinness of the freedom that surrounds the condition of temporary agricultural workers was thus being discursively nominated, too.

An instance of agricultural workers’ protests took place in July in Albacete, close to my native town. In that generally uneventful provincial capital, a group of workers left the settlement where they were confined to quarantine after positive COVID-19 cases had emerged in their working group. They rallied into the city center, chanting demands for both improved working conditions and the protection of their right to work (Vecina 2020). Reports on these protests contributed to make publicly visible that these workers were living in “illegal” makeshift quarters with insufficient equipment and lacking basic services, many of them sleeping on the floor and without access to toilets or showers—a situation that had been denounced every summer, during the harvesting season, for over a decade, without effect. Despite those conditions, and in some cases despite illness, those workers complained about the confinement because they did not want to give up their jobs. Their position was, needless to say, clearly distinguishable from the arguments of those who oppose government-fostered confinement in the name of abstracted notions of individual liberty: “if they do not work, they do not eat,” summarized a local member of a felt-leaning party in support of the worker’s demands (ibid.). That basic statement rings accurate when assessed in relation to the actions those workers undertook those days: so much is at stake for them in a day’s work wage that they chose a Sunday to go on their rallying protest—that is, the only day most of these workers cannot earn a wage even if they wanted to (it is the day off work).

It became clear that these “essential workers” do not see quitting the job as an option. Elsewhere, commenting on the risks associated with searching for seasonal work in the agricultural fields of Lleida (where COVID-19 infection rates had been very high), a Senegalese worker stated: “we are poor, we do not fear death or sickness, we fear misery” (Congostrina 2020a [my translation]). In yet another different region but in an equivalent situation, another explained: “I don’t fear coronavirus. What terrifies me is not to be able to feed my three children” (Congostrina 2020b [my translation]).

The position of most workers in the public health system is vulnerable too, but marked and appeased by different conditions. For them, losing a job is of course catastrophic in many ways, but if that happens, and leaving other factors aside (like the likelihood of pre-existing local networks of support or of monetary savings), they can generally count on some initial safety nets: temporary unemployment assistance and, occasionally, some other public benefits. But these workers are also experiencing growing labor precariousness, and intense stress and exhaustion have settled among them, to the extent that some have actually quit their jobs in this period (Plaza Casares 2020b).^3^ A proportion of them move on to get employment abroad, in other countries of the EU with better salaries and working conditions for employees in their sector (in the year before the pandemic there had already been a 20% increase in requests of accreditations to work abroad, indexing widespread discontent in the sector [ibid.]); others stay in the country, searching for employment outside the public health system. And in any case the number of voluntary redundancies would be probably much higher if employment options were not limited by structural factors—the so-called labor market in Spain is relatively narrow even for qualified health professionals (with considerably high levels of unemployment) and insecure (with high levels of precariousness in the work place). This seems to shape their thinking about the possibility of quitting jobs too, unsurprisingly.

This was well illustrated in an op-ed piece in a Spanish newspaper in which a doctor based at a health center in Madrid summarized how some of his colleagues explain what keeps them working despite the difficult conditions experience during the pandemic. Most of them placed emphasis on vocational commitment, on service to community and on the humane dimension they realize through their jobs. But vocation shows some material limits among those who have to make a living out of their labor, doctors among them: “the principal [reason why I keep working] must be that I have nowhere to go [for work],” one doctor remarked (Serrano Morón 2020 [my translation]). Probably anticipating moralizing critiques from sectors of the readership, the author of the op-ed felt obliged to remind readers that doctors (like any other workers who can afford a deposit to buy a house in Spain, one may add) have heavy mortgages to pay back. For those of them coming from working class families or with no previous family assets to rely on, their access to housing fully depends on market mechanisms that continuously threaten the right to housing even to those with “middle-class” employment.

These glimpses into “essential work” in Spain provide multiple avenues to understand how the social process we encapsulate under the term “work” shapes individual and collective experiences and behavior. “Work” throws punches that are difficult to side-step, and this helps explain why this abstraction has been central to different streams of social theorization for more than two centuries. This abstraction soon became a spring for such theorization in societies undergoing the transformations that the emergence of “modern society” (the society of capital) brought about. Everyone had to make sense of those transformations as they lived through them, and scholars in particular aimed to explain them with the tools of newly emerging paradigms of social-scientific production. In the name of studying “the economy,” those scholars started to work out a labor-based theory of value so they could address questions that were being collectively and conflictively shaped amidst the social motion of their times. The labor theory of value took on different directions, and to this day remains a contested terrain of analysis.^4^ Yet, in all cases, grounded on the basic abstraction of “work” whose social manifestations I have been exploring with a focus on the category “essential work” in Spain, it enabled the generation of new questions about society, human behavior, and meaning production.

After the interventions of Marx, Engels, and those who contributed to the variegated Marxian corpus of critical scholarship, this theory also facilitated the generation of additional questions about history, cultural production, and power. These were, and continue to be, anthropological questions, and the scenario created by the COVID-19 pandemic provides a propitious opportunity to reflect on why that is the case—it has brought out the child that everyone has inside.

This special issue seeks to stimulate that reflection on how the labor theory of value can creatively inform and reformulate in critical ways a variety of social issues addressed through anthropological lenses—issues that range from Bitcoin production to the new agricultural “digital revolution” to tourism in the semi-peripheries of the world system (see Fiona McCormack’s article in this issue for detailed commentary on these approaches). By doing so, this issue aims to enliven key debates within the discipline, and in what follows I situate these debates specifically in relation to the potential utility of the labor theory of value for anthropology. First, I provide a brief historical overview of the foundations of the labor theory of value, outlining the reasons why it opened new grounds for anthropological and, more generally, for social-scientific enquiries—not only for those interested in “the economy.” Second, I discuss key points of friction between scholarship that attempts to develop an “anthropological” theory of value as an end in itself and anthropological scholarship that resorts to the (labor) theory of value to critically inform research—a friction that resonates with old, and important, theoretical debates within the discipline. Finally, I outline some ideas about the ongoing relevance of the labor theory of value for the anthropological task.

## The labor theory of value, Marxian critique, and anthropological enquiry

Labor theories of value were already a fundamental pillar in the study of capitalist society by the time Marx embarked on his own scrutiny of its logics, pace a critique of classical economists (Collins 2016: 105–106; Angosto-Ferrández 2016: 3–5). Adam Smith had situated labor at the center of his seminal theorization of national wealth (2012 [1776]), recasting the conceptualization of its productive powers and aiming to set a frame for analyzing the social mechanisms that enhance or limit those powers, in benefit or detriment of his view of common good. Smith conceptualized a link of dependence between (exchangeable) value and labor, identifying the latter as the source of the former. In logical accord with that conceptualization, he also contributed to demarcate new categories for socioeconomic analysis. An analytically pivotal one was a distinction between groups of people that undertake “useful labor” and those that “do not labor at all” but can nonetheless consume far more than most direct producers (ibid.: 3–4). Social class as analytical category was pre-figured in those efforts—leaving aside any political or moral assessments that such categories could generate within the theoretical terrain demarcated by Smith.

Ricardo (2001 [1817]) sharpened those analytical categorizations. Avoiding “inconsistent language,” as he put it, he provided more conceptual rigor to the theorization of labor as the substance that generates (exchangeable) value. His work enabled more refinement in the analysis of the tensions and balances generated among groups of people that, through their specific position within a determined set of social relations, receive a share of realizable value—a share that does not necessarily keep proportional relation with their involvement in its generation. The mechanisms of distribution of value received more systemic scrutiny from Ricardo’s perspective, but the relations (including property relations) that condition their production could not be ignored. Social class thereby gained additional potentials as an analytical category.

Marx benefitted from that preceding stream of scholarship when embarking on *Capital*. His own approach to theorizing value, and to exploring the configuration of the inter-dependence of the social classes among which the total value of a society’s production is asymmetrically distributed (“the trinity formula”), was indeed in direct dialog with elements of that classical scholarship. But those classical contributions were recast under critical perspectives: Marx set out to demonstrate why economists had little of substance to say about the logics of Capital (as a social relation) if they conformed themselves with rationalizing economic process as if it were a natural process.

Specifically in relation to the labor theory of value, Marx followed classical economists in maintaining the conceptual link between the powers of labor and the creation of value for the study of the “laws of motion” of Capital. But in doing so he pursued goals that were not only technical, but also epistemological: overcoming a narrow focus on “the magnitude” problem that absorbed the efforts of many economists (Marx 2008: 483, note 1), Marx aimed to provide an integral, and historically situated, characterization of those laws so the search of what he envisaged as liberating alternatives to such laws could be enabled (Fernández Liria and Alegre Zahonero 2011). This effort included a reconsideration of the historical conditions that had facilitated the emergence of capitalism as a social form: through a revision of the question of “primitive accumulation,” the role of unequal social power and violence were brought to the fore (Perelman 2000; Angosto-Ferrández 2020: 4–6).

The ideological drives that shape behavior in capitalist societies, often normalized as natural human traits by classical political economists, were as a result destabilized, as was the conceptualization of the human condition that underpinned the theorization of those economists (Patterson 2009: 41–50): from the perspective opened by Marx, people’s behavior, beliefs, and cultural creations could be read as historically and socially conditioned, and not as the realization of a universal and a-temporal human essence.

As a whole, Marx’s scrutiny of the logics of Capital contributed to affirm an epistemological turn that, beyond political economy, maintains a presence in the social sciences to this day. Even those with no interest in transforming the world were offered new theoretical grounds to explore the relations between the “outward appearance” and the “essence” of things against the background set by that general abstraction that the labor theory of value underpinned (1972 [1894]: 817). “Work,” understood both as that fundamental process that makes social life possible and as a realm that shapes and is shaped by historically determinate conditions and social relations, became a reliable compass (rather a pre-established answer) for “essential” analysis among those approaching the study of social issues with an interest in providing explanation and searching for causalities. From this perspective, the relations between social formation, meaning production, and culture writ large could be examined as a part of a holistic system that was historically contingent, but which nevertheless maintained a central locus of systemic configuration in “work” as social process.

In parallel, Engels joined Marx’s explicit efforts to enhance the anthropological potential of this perspective. Against the analysts that approached “labor” as a mere technical concept for the analysis of value creation (on occasion in the name of Marxist theory), and drawing from the contributions of Darwin and other evolutionary theorists, Engels (e.g., 1950 [1876]) aimed to summarize arguments about why labor is “infinitely more” than the spring of wealth to which orthodox economists referred to.

Building on the idea that labor was the spring of human corporeal and social organization, central to Marx’s anthropological thinking too (Patterson 2009: 44–46), Engels approached labor as the linkage between anthropogenesis and sociogenesis (cf. Fluehr-Lobban 1986). Labor was anthropogenetic because human “organisms” (and human selves) had developed in relation to the activities it entails. But these activities were also sociogenetic, since it was through the increased interactions that labor activities spurred that language and speech developed—in turn a new engine for intensified and more complex forms of sociality.

In that same effort, Engels (1950 [1876]) outlined a critique of idealism (as a theoretical perspective) that also flagged the centrality of labor as a causal force in changing cognitive capacities and in shaping conceptions of the world. It is worth remarking this point in light of the complex dimensions that, beyond the relation with value production, Engels and Marx associated with labor as generative process. Both had already spent decades building (and publishing on) their philosophical and political critique of idealism, ranging from earlier philosophical assessments of Hegelian dialectical idealism to the polemics with orthodox economists and with utopian socialists. This critique had been a spring of theorization that enabled them to better demarcate the contours of their historical materialist perspectives. But with the focus on labor as developmental engine and as a locus of cognitive transformations, Engels was bringing, in dialog with the scientists of his time, an empirical, anthropologically grounded hypothesis to explain the generation of idealism as a perspective on the world. In simplified form, the argument was that, as societal complexity increased, the very same gradual development of cognitive skills that had accompanied anthropogenesis (underpinned by the labor process) would have facilitated an “idealistic world outlook” to settle among people. From such a perspective, the examination of worldviews and ideological formation could remain anchored in the examination of the materially grounded social process underpinned by labor.

Varieties of these perspectives have informed the work of anthropologists and other social scientists that cultivate what nowadays we identify, in very general terms, as evolutionary anthropological streams. These streams of scholarship parted from other approaches to the anthropological task, in processes of intellectual divorce that gradually crystallized in firm institutional entrenchments: the divides between biologically/ecologically oriented and socioculturally oriented anthropology departments have grown ever more marked in the past decades, with scholarly dialogs between their respective members remaining a rarity. However, the epistemological perspectives that pervaded Engel’s work also remained influential, even if often in latent form, for social anthropologists. That influence is reflected in anthropological perspectives that do not detach the study of society, culture, or behavior from an understanding of the forces generated by that abstraction we denominate “work.”

It is not my intention to provide here an overview of these perspectives, or to discuss their internal differences or the theoretical features that separate them from other perspectives in anthropology. Recent research has provided valuable commentary in this respect (Narotzki 2018; Neveling and Steur 2018; Harvey and Krohn-Hansen 2018; Kalb 2015; Nugent 2007). Yet it is additionally worth recalling that engagement of Marxian theory in mainstream academic institutions has gone through fluctuations that cannot be merely associated with the strength that anthropologists may find in alternative theoretical perspectives when approaching the object of their studies.

For instance, Marxism occupied a place in the anthropology university curriculum in core countries of the capitalist bloc in the aftermath of Second World War and during the first stages of formation of the Cold War. In the Europe within that sphere, the gradual opening of academia to working class students and the general ideological tone of the postwar political consensus was in the background. That tone still resonated with conceptualizations of social and economic rights as human rights that, under the influence of the Mexican and Soviet revolutions, had been incorporated into that consensus as mechanisms to sustain an inter-class *entente cordiale*. Heterodox, Keynesian takes on the regulation of market economies were on the rise and facilitated a larger degree of intellectual openness in academic institutions too. But these conditions were transformed as the ideological wars that accompanied the Cold War entrenched—scholarship became also a business to be undertaken in “blocs.”

This of course affected anthropology departments in core countries such as those in the UK and other European institutions, but was also sharply noticeable in the USA, which, at the same time as becoming the main engine of globalizing academic production, developed a system of open or timidly covert persecution of scholarship that governments and intelligence agencies labeled as subversive—which within anthropology did not only include scholarship considered to be influenced by “communism” through Marxian theory, but also the work of anthropologists who were active advocates of racial equality or anti-fascism (Price 2004).

At any rate, Marxian theory has provided orientation to anthropologists that see its continuing relevance even when adopting critical stances or reconsidering some of its foundations. It is worth remarking the strength of recent research that maintains an anthropological dialog with “work” as an abstraction, whether directly with a focus on labor forces and processes or through other avenues like those associated with the study of the impacts of property as a social relation (e.g., McCormack 2017; McCall Howard 2017; Kasmir and Carbonella 2014).

That position of critical dialog is markedly distinguishable from streams of anthropological research that pursue what they consider a distinctively “anthropological” theory of value while fully disregarding (or misrepresenting, when they regard it) the anthropological grounds of the labor theory of value.

## Critical takes and rejections: current anthropological approaches to the labor theory of value

The reason why dialogs with the labor theory of value have been abandoned by those who pursue a distinguishable theory of value for anthropology remains unclear, since this has occurred without much discussion of its limitations or potentials. Indeed, Marxian contributions are often misrepresented or totally neglected within the renewal of approaches to theorize and study value as an analytical concept in anthropology. This is well illustrated by the work of David Graeber (2001), which remains an influential reference point in that renewal (see also Pedersen 2008).^5^ When justifying his theoretical pursuit, Graeber’s (2001: 1–2) characterization of the way in which value had been defined “in the economic sense” fully displaced Marxian contributions to the debate—and, more broadly, Marxian contributions to social theory. Value in the “economic sense” was in that view something defined in the sphere of exchange, detached from the conditions and mechanisms that, from Marxian perspectives, generate and shape the distribution of value in the first place. The productive powers of labor and the social relations of production that condition and articulate it, including property relations, vanish from the scene of analysis.

In intellectual exercises that separate meaning production from materially grounded social process, “value” is uprooted from any anchorage it might have within labor theories, and is instead located in a theoretical terrain in which value becomes a term that encapsulates conceptions of the desirable. Varieties of Kluckhohn’s (1951) definition of (social) values pervade these perspectives, which transform “value” into a concept that, as far as the theorization of society goes, reproduces notions of societies (or “cultures,” to use a favored term from these perspectives) that bind people and provide meaning to their actions through sets of organizing principles that “value” would articulate (Angosto-Ferrández 2016: 8–15).

These theories do not provide tools to question the origin of those bounded spheres of meaning production (“cultures”), nor to explore the forces that may generate social change (or change in “values”)—unless one accepts that these social aggregates we denominate cultures are self-generative, and furthermore that changes in “values” are caused by the strength of autonomous ideational production. From these perspectives, discussions of inequality end up detached from examinations of social process as this is partly shaped by “work.”

In contrast, other streams of anthropological research continue to pursue conceptualizations of value as drivers of social analysis critically linked to labor theories. That stance has precluded dogmatic reifications of the theory, which is brought to respond to critical challenges and to ongoing societal transformations. As authors such as Collins (2016: 104) have noted, potential Marxian contributions to the analysis of contemporary capitalism can for instance be fruitfully revitalized with critiques presented from feminist and environmentalist fronts, without this implying a rejection of the labor theory of value.

Collins own work provides an example of this approach with her conceptualization of value in relation “to the material limits of a particular societal division of labor” (ibid.: 121). Infusing her analysis with feminist scholarship, she proposes to overcome narrow readings of value as labor, but does so without uprooting the conceptualization of value from the social process of material production (Collins 2016: 120). This enables her, among other things, to theorize the role that the state plays (or could play) in guaranteeing social reproduction in societies in which labor markets, and contemporary capitalism writ large, have destabilized and reorganized, in atomistic forms, the localized networks that previously provided the grounds for that social reproduction—even if arguably at the expense of a gendered division of labor in which women became the source of non-salaried and politically subordinated labor. Many people thus come to rely on this public authority we denominate the state as the source of support for the provision of forms of care that otherwise they cannot provide anymore, since they are only accessible through market mechanisms that, in practice, exclude them from the possibility of “buying” the service of care. The dramatic human cost of that type of exclusion is easy (if painful) to understand, and to approach in boldly sentient forms—think of a person you feel or become responsible for (be it for social and/or elective affinities [a parent or a partner, a sibling or a friend, a son/daughter or a neighbor]) turning destitute. This helps explain why and how people organize to undertake collective action in demand of the rights of state workers (Collins 2017), which are in this light a key to defend their own rights (and affects).

## Final comments

The labor theory of value is social theory, not a theory about “the economy.” And, as any other stream of social theorization, that theory speaks to us in anthropological terms. Social theory is necessarily mediated by analytical conceptions. These may become elaborately formulated by experts, or may remain tacitly applied onto research agendas; but in either case they are conditions of theorization of the social and set epistemological boundaries to the process of knowledge production.

These analytical conceptions include and (re)shape ideas about what makes us human, what is a person, what is society, what is culture, what drives and shapes individual and collective behavior. Social theories combine and articulate those conceptions in frames of analysis and explanation that, in their capacity to produce knowledge about the social world, are simultaneously enabling and limiting: they enable us to pose (and potentially answer) certain questions about what people are and what people do, and why so; but those frames are also limiting because they conceptually impede (or reject) the formulation of other questions about what people are, what people do, and why so.

In this paper I have tried to show how, and why, the questions that the labor theory of value enabled us to explore and potentially answer remain anthropologically relevant. Initially I did so through an examination of the social dynamics that the current pandemic has generated in Spain, and subsequently I aimed to clarify why it is not only in times of marked social crisis that this theory is applicable onto anthropological research. A historical overview of the Marxian interventions in the labor theory of value underscores that this theory opened paths to new forms to examine, and to potentially explain, the relations between social formation, meaning production, and culture writ large. Beyond specific social conjunctures or territorial boundaries, those contributions have been, and remain, a fruitful and often enlightening ground for anthropological research. This issue illustrates this point: anthropology as a discipline continues to have much of importance to say about people’s lives when it maintains its concern with understanding those lives and people’s creations in relation to the social processes that enable and limit them.

## Data Availability

Not applicable.

## Footnotes

1 For an overview of the way in which the category “essential workers,” or “essential” labor, is being differently interpreted by US workers seeking to organize amidst the COVID-19 crisis, see Kasmir (2020: 309–310).

2 These difficulties are not only internal and due to the temporary character of this work, the legal status of these workers (often immigrants on temporary visas) and fears of reprisal, but also, as some authors note, because of the disdain with which some established unions treat these foreign workers who can easily be deported or replaced, and which therefore rarely can use the threat of withdrawing labor as a bargaining strength. See Weiler and McLaughlin (2019: 381–382) for a discussion of this case in Canada.

3 This was particularly marked in positions in which the lack of adequate resources turned their work in what they view as a growingly alienating and inefficient task. For instance, some doctors in the area of Primary Assistance and family doctors have been working under a regime in which they can only dedicate 6 min to each of patient if they want to meet their targets (see https://www.elsaltodiario.com/sanidad-publica/sanitarias-renuncian-puesto-atencion-primaria-no-se-puede-ejercer-asi).

4 The historical prominence of this contest cannot be overstated. The “right” interpretation of labor theories of value has been, and remains, a point of definition of radically divergent perspectives on social theorization, far beyond the realm of theorization of “the economy.” Nothing more revealing that the efforts that orthodox economist and liberal economists dedicated to produce non-Marxian readings of David Ricardo and Adam Smith, aiming to negate any interpretation of their understanding of the conflict between wages and profit as a foundation of a class antagonism (e.g., Tucker 1961).

5 Let me present these critical notes as a show of appreciation toward Graeber’s work, since I am writing a few months after his passing away.
